# Prion Protein Misfolding

**DOI:** 10.2174/156652409789105543

**Published:** 2009-09

**Authors:** L Kupfer, W Hinrichs, M.H Groschup

**Affiliations:** 1 Department of Production Animal Health, Faculty of Veterinary Medicine, University of Calgary, 3330 Hospital Drive NW, Calgary, Alberta, T2N 4N1, Canada; 2 Institute for Biochemistry, University Greifswald, Felix-Hausdorff-Str. 4, D-17489 Greifswald, Germany; 3 Institute for Novel and Emerging Infectious Diseases, Friedrich-Loeffler-Institute, Federal Research Institute for Animal Health, Südufer 10, 17493 Greifswald-Insel Riems, Germany

**Keywords:** Transmissible spongiform encephalopathy, prion protein, structural propensities, misfolding.

## Abstract

The crucial event in the development of transmissible spongiform encephalopathies (TSEs) is the conformational change of a host-encoded membrane protein - the cellular PrP^C^ - into a disease associated, fibril-forming isoform PrP^Sc^. This conformational transition from the α-helix-rich cellular form into the mainly β-sheet containing counterpart initiates an ‘autocatalytic’ reaction which leads to the accumulation of amyloid fibrils in the central nervous system (CNS) and to neurodegeneration, a hallmark of TSEs.

The exact molecular mechanisms which lead to the conformational change are still unknown. It also remains to be brought to light how a polypeptide chain can adopt at least two stable conformations. This review focuses on structural aspects of the prion protein with regard to protein-protein interactions and the initiation of prion protein misfolding. It therefore highlights parts of the protein which might play a notable role in the conformational transition from PrP^C^ to PrP^Sc^ and consequently in inducing a fatal chain reaction of protein misfolding. Furthermore, features of different proteins, which are able to adopt insoluble fibrillar states under certain circumstances, are compared to PrP in an attempt to understand the unique characteristics of prion diseases.

## INTRODUCTION

The Prion Protein (PrP) belongs to the class of amyloid-forming proteins which are, in some cases, associated with certain diseases. The cellular prion protein (PrP^C^) is a membrane associated protein occurring in a wide range of eukaryotic cells. The wide distribution among mammalian species and the high conservation of PrP^C^ indicates a role of general importance. However, the physiological function of PrP^C^ is still unknown. 

According to the ‘protein-only’ hypothesis [1], PrP^C^ is able to undergo a conformational transition into an insoluble isoform known as PrP^Sc^ (‘Sc’ for ‘scrapie’) and which is thought to be the agent that causes transmissible spongiform encephalopathies (TSEs). TSEs are fatal neurodegenerative diseases, including among others Creutzfeldt-Jakob-Disease (CJD) in humans, scrapie in sheep and goats, as well as bovine spongiform encephalopathy (BSE) in cattle [2, 3]. It is the transmissibility of prion disorders which distinguishes TSEs of other protein misfolding diseases. There is evidence that the decisive process i.e., the irreversible conversion of the physiological membrane associated cellular prion protein (PrP^C^) into its disease-related proteinase K (PK) resistant counterpart PrP^Sc^, initiates an ‘autocatalytic’ reaction which leads to the accumulation of amyloid in the central nervous system (CNS) and, through still unknown mechanisms, to neurodegeneration [4, 5].

A noteworthy, and heretofore unexplained, characteristic of TSEs is the existence of different strains, which can be distinguished due to specific incubation times and clinical signs *in vivo* [6] as well as by distinct biochemical or immunohistological characteristics *in vitro* [7-9]. It is still unknown how strain-specific characteristics are supposed to be transmitted by a protein itself. Structural determinants such as glycosylation are thought to be involved in strain-dependent specification of PrP^Sc^ structures and are a characteristic distribution in affected brains [10, 11]. However, the occurrence of prion strains and the protein-only hypothesis have not yet been reconciled. Despite proceeding findings in prion research, the exact mechanisms that underlie the conformational change or conversion of PrP^C^, as well as those that cause the typical pathological pattern of TSEs, remain an enigma [2, 12]: hence, the development of rational approaches to diagnosis and therapy are restricted [13-15]. 

The feature to undergo induced or spontaneous misfolding was shown to depend on structural aspects of PrP^C^, such as the amino acid sequence [16-19], the highly flexible amino terminal region of the protein [20] as well as secondary structure elements [21, 22] and posttranslational modification elements [11, 23]. The remarkable peculiarity of PrP to adopt several structurally favourable states requires a detailed contemplation of distinct structural parts of PrP^C^ and their possible role in PrP^C^-PrP^Sc^ interaction, misfolding and disease transmission. Cofactors, like metal ions [24, 25] or proteins [26, 27], are also thought to be involved in the structural determination of PrP, but the manner in which they influence structure and interaction with other molecules is yet to be determined. Furthermore, whether they have an effect in preventing prion protein misfolding is also in question. 

The purpose of this review is to highlight different sections of PrP^C^ and their possible role in PrP^C^-PrP^Sc^ interaction and prion protein misfolding. Additionally, features of other proteins that are able to adopt insoluble fibrillar states under certain circumstances, are compared to PrP with regard to our understanding of the unique characteristics of prion diseases.

## STRUCTURE DETERMINATION FOR PrP^C^

The molecular structure of PrP^C^ at atomic resolution has been determined by nuclear magnetic resonance (NMR) spectroscopy and X-ray crystallography. PrP^C^ consists of a long and flexible amino terminal region spanning up to amino acid (aa) residue 121 and a structured carboxy terminal domain. This globular domain harbours two short sheet-forming anti-parallel β-strands (aa 128 to 130 and aa 160 to 162 in murine PrP^C^) and three α-helices (helix I: aa 143 to 153; helix II: aa 171 to 192; helix III: aa 199 to 226 in murine PrP^C^) [28]. The length of the unprocessed translation product is 256 amino acids. In the course of its transit through the ER and Golgi apparatus, post-translational modifications occur, such as the removal of a N-terminal signal sequence (1-22); the formation of an internal helix II and III stabilizing disulfide bond (between aa 179 and aa 214); the attachment of N-linked oligosaccharide chains (at aa 180 and aa 196); and the replacement of the carboxy terminus (at aa 231) by a glycosylphosphatidylinositol (GPI) anchor [29]. Fully processed (murine) PrP^C^ therefore contains only 209 amino acids, representing codons 23-231 of the prion ORF. The glycosylation can be missing or occur at either one or both sites so that cells harbour non-, mono-, as well as diglycosylated isoforms of PrP^C ^(Fig. (**1**)).

## MODEL STRUCTURES OF PRP^SC^

In contrast to PrP^C^, the three-dimensional structure of PrP^Sc ^has not yet been fully established, since this protein could not be purified in sufficient quantity and quality in a soluble, non-aggregated form.

Amyloidal fibrillar structures are characterized by tinctorial assays using Congo Red and Thioflavin T, far ultraviolet CD-spectra which identify β-structures and typical anisotropic 'cross-β' X-ray diffraction pattern. X-ray diffraction studies of amyloid fibres have shown that their protofilament cores all contained a 'cross-β' scaffold in which β-strands are arranged perpendicularly and β-sheets are parallel to the axis of the fibre. The stabilization of the core structure is basically provided by hydrogen bonds and other interactions, including the polypeptide main chain. Some amino acids at certain residues may support the process of arranging fibrillar structures. An alternation of polar and hydrophobic residues can result in a formation of the same kind of β-sheet structures that are found in amyloid fibrils [30]. Crystal structure analysis of short oligopeptides, which are parts of amyloid-forming proteins, revealed that segments of four to seven amino acid residues are sufficient to form fibrils. The assumption is that amyloid-like fibrils are formed by two tightly interdigitating β-sheets in a zipper-like manner, allowing nucleation to fibril forming aggregates. Alternately, this process can also start by the unmasking of short zipper-forming segments, which then stack into β-sheets.

However, on the basis of cryoelectron microscopy and by the means of structural modeling based on similar common protein structures, it has been discovered that PrP^Sc ^contains ß-sheets in the region of aa 81-95 to aa 171, while the carboxy terminal structure is supposedly preserved. These ß-sheets form a left-handed beta-helix. Three PrP^Sc ^molecules are believed to form a primary unit and therefore build the basis for the so-called scrapie-associated fibrils [31] (Fig. (**2**)).

The increase in the content of β-sheet structures results in insolubility in mild detergent fluids and causes partial resistance to enzymatic degradation of the pathogenic isoform PrP^Sc^. If PrP^Sc^ is treated with proteolytic enzymes, only the N-terminal amino acids (aa) up to residues 81-95 (depending on the TSE agent and the proteolytic conditions) are digested [32, 33], leaving the remaining PrP^Sc ^reaching from aa 81-95 to aa 231. The increased resistance to proteolysis leads to an accumulation of PrP^Sc^, which can be made visible by special amyloid or immunohistochemical staining. PrP^Sc ^is also deposited outside the cell as well as in the lysosomal-endosomal compartments within the cell [34]. The disease-causing agents are PrP^Sc^ aggregates that act as templates for the conversion; its catalytic activity depends on the size of the particle. PrP^Sc^ particles which consist of only 14-28 PrP molecules exhibit the highest rate of infectivity and conversion [35].

## PRION PROTEIN MISFOLDING DISEASES – A COMMON PATHOLOGICAL PHENOMENON 

Apart from prion diseases, there are a number of other protein misfolding diseases: Alzheimer’s disease, Parkinson’s disease, Huntington’s disease, spinocerebellar ataxias, type II diabetes, amyotrophic lateral sclerosis, as well as diffuse Lewy body dementia and the fronto-temporal dementias. Studying the key molecular mechanisms involved in prion diseases may also help to understand these other amyloidoses. Inducible proteinopathies, such as amyloid A amyloidosis or apolipoprotein A II amyloidosis, show remarkable similarities to prion diseases [36]. Most recently Meyer-Luehmann [37] reported that in Alzheimer's disease the exogenous induction of cerebral ß-amyloidogenesis is governed by agent and host factors. The striking parallels of infectious prion disorders to the above-mentioned putatively non-infectious protein misfolding and assembly diseases make it more and more difficult to delimitate their pathological mechanisms from each other. 

Of all the proteins known to undergo misfolding, the prion protein has been, and will likely continue to be, one of the most thoroughly researched. These investigations targeted the structural stability of PrP^Sc^ and PrP^C^, or they examined the propensity of PrP^C^ to be folded into PrP^Sc^, using cell-free systems, infected cell lines or transgenic mice. Structural stability studies on PrP^Sc^ include partial or consecutive protein denaturation steps by urea or guanidinium; subsequent measurements include using a conformation dependent immunoassay (to determine the epitope accessibility to antibodies) or circular dichroism spectra. Similar experiments have also been carried out to determine the unfolding characteristics and structural stability of PrP^C^. However, PrP^C^ conversion assays are the most frequently used approaches and include a large variety of different experimental setups. 

Protein folding and hence misfolding is determined by the primary structure of a polypeptide chain, but the complex process of protein folding kinetics has been a major topic for decades and is still not completely understood. Despite numerous models for protein folding, there also exist various theories as to how misfolding could be explained. 

It is likely that PrP^C^ undergoes some intermediate states of structural organisation before finally ending up as part of an amyloid fibril. Misfolding can only take place when the native structure of a globular protein is at least partially unfolded or degraded. Beside the prion protein, there are numerous other soluble proteins which can self-assemble to an amyloidogenic state and, as far as is known, display similar features concerning their structure [38]. 

A certain tertiary structure of a protein represents the equilibrium of the polypeptide molecule with the chemical environment, defined by the surrounding solvent, salt concentrations and pH. The overall folding stability, i.e. the free energy term of globular proteins is in the range of one or two hydrogen bonds which allow the transition to alternative conformations in the energy landscape without the threshold of high transition energies. As a result, for several proteins, alternative conformers are now known [39, 40]: rapid refolding under physiological conditions has been shown for spider-silk proteins that form β-sheet rich fibres contingent upon the rapid decrease of sodium, increase of potassium concentration and a drop in the pH (8 – 6) [41, 42]. If buffer conditions are changed for the all helical apo-myoglobin, then β-strand containing, fibre-forming aggregates occur [43, 44]. The authors suggest that cross-β-conformation is dominated by protein main chain interactions common to different polypeptides, whereas specific side chain interactions will define the characteristic main chain fold of globular proteins. Furthermore, they conclude that evolutionary adaptation, including mutational sequence variation and molecular chaperones, suppresses amyloid formation of globular proteins *in vivo*. Fibril formation of PrP can also be initiated by certain buffer conditions without the requirement of an infectious PrP^Sc^ seed [45]. Spontaneous protein misfolding may occur more frequently under physiological conditions than is generally assumed. Cellular factors and pathways could be of major relevance in regards to disease prevention or initiation.

Chaperones may have a key role in preventing pathogenic effects of misfolding and aggregation. An interesting example is the extracellular chaperone clusterin, which inhibits amyloid formation of human lysozyme [46]. Clusterin interacts with oligomeric prefibrillar species, which are present in the nucleation phase prior to aggregation. Apparently, these interactions support the dissociation of the prefibrillar intermediates into native monomers. As for PrP, chaperones have been shown to play an interchangeable role: certain heat shock proteins are able to promote conversion, whereas others inhibit misfolding [47]. The chaperon BiP, which is present in the endoplasmatic reticulum (ER), has been shown to bind to certain forms of PrP that were retained in the ER due to incomplete processing [27]. Within the ER, BiP is believed to maintain proper folding of PrP by binding to defective forms for an extended period of time. In this way, the defective forms can finally be degraded by the proteasomal pathway. 

Due to their occurrence in amyloids, there is evidence for the assumption that nucleic acids, lipids and glycosaminoglycans (GAGs) might play a role as cofactors in amyloidogenesis. For this reason they could be a useful therapeutic target not only for prion disorders but also for other protein misfolding diseases.

The significant effects of possible cofactors have been demonstrated in numerous experiments. In an interesting study by Yin and colleagues [48], it was shown that recombinant PrP harbouring different pathogenic mutations had a more exposed amino terminus and bound more strongly to glycosaminoglycans. As common components of amyloid [49] GAGs are found in PrP^Sc^ *in vivo* [50], it has been shown that they facilitate the conversion of PrP^C^ into PrP^Sc^ *in vitro* [51], as well as PrP-aggregation [48]. Lipids and nucleic acids also bind to PrP^C^ and are detectable in PrP^Sc^-aggregates [52-54]; additionally, they may facilitate PrP-conversion by functioning as a scaffold that binds and concentrates PrP^C^ in order to provide high amounts of substrate for a conversion into PrP^Sc^.

## EFFECTS OF THE PRIMARY STRUCTURE OF PRION PROTEIN ON ITS MISFOLDING PROPENSITY 

In light of recent studies, significant differences among different species concerning the tertiary structure of PrP^C ^are unlikely due to a high degree of structural and organizational homology between mammalian PrP sequences and structures [55]. However, there are indeed major associations between the convertibility of the various PrP^C^s and the variability of single amino acids at certain positions within PrP^C^. Single amino acids at certain positions of PrP^C^ can have striking effects in relation to either the susceptibility to TSEs or the chance to develop inherited forms of human prion diseases. There are more than twenty mutations of the prion protein gene *(prnp)* that are known to be associated with or that are directly linked to human TSEs [56]. A well known polymorphism in the human PrP gene is located at codon 129, which either encodes for methionine or valine: it influences the susceptibility to sporadic [57] or acquired TSEs [58], as well as the age of onset of the disease [59]. It was demonstrated that this polymorphism even has an impact on some misfolding pathways in a cell-free conversion assay which was described by Baskakov [19, 60]. This assay does not initiate the folding reaction by co-incubation with PrP^Sc^ but uses certain buffer conditions that result in different types of PrP formation. Choosing an alternative pathway with partially folded human PrP allelomorphs, the valine consisting ones showed less delay in amyloid formation compared to the methionine allelomorphs either under spontaneous or seeded folding conditions. Rezaei and colleagues [61] performed unfolding experiments using different variants of ovine PrP. Polymorphisms in the sheep PrP affected thermodynamic and kinetic parameters of the unfolding as well as the refolding process. The results of the experiments indicate a molecular basis for the effects of PrP polymorphisms on the transformation of PrP^C^ to PrP^Sc^.

Several other *in vitro* conversion experiments also showed the correlation between the PrP amino acid sequence and the convertibility of PrP^C^ into PrP^res^. Depending on the genotype, ovine PrP was converted into its PK-resistant counterpart in a cell-free assay, using sheep PrP^Sc^ as seed for the conversion reaction [62]. Ovine PrP genotypes, which are not susceptible to classical scrapie *in vivo* also failed to be converted to PrP^res^ *in vitro*. Another cell-free assay in which bacterially expressed PrP^C^ was co-incubated with PrP^Sc^ from mouse scrapie brains [63] was used to demonstrate that a single amino acid substitution within a mouse-ovine chimeric PrP^C^ results in an inconvertible mutant of the previous convertible molecule [64]. 

The aforementioned matters in regards to the PrP amino acid sequence raise the question of how a single, or simply a few, amino acid residues at certain sequence positions can have such striking effects on the convertibility without changing the globular structure of PrP^C^. Basically, two types of PrP conversion can be distinguished – induced misfolding and spontaneous, or non-seeded, PrP^Sc^ or PrP^res^ forming. The latter is seen in inherited human TSEs, whereas the induced misfolding needs an infection to begin, e.g. through the oral intake of infected tissues. The misfolding kinetics of both processes can be similar. As indicated by experiments *in vitro*, an initial lag phase is followed by a growth period of rapid fibril formation [65]. The addition of prion particles during the lag phase shortens the same and is known as seeding. A physiological equilibrium of PrP^C^ and PrP^Sc^ could be destabilized by either exogenous infectious particles (acquired TSE) or a high amount of spontaneously misfolded endogenous PrP^Sc^ (spontaneous disease). The incubation period or the time until the onset of the disease therefore depends on the stability of this equilibrium. Certain amino acid constellations tend to induce the misfolding more often than other sequences and subsequently lead to fibril formation as seen in susceptible PrP genotypes.

It has been demonstrated by NMR spectroscopy that some disease related mutations of the human PrP^C^ are located in a part of the protein that is involved in the maintenance of the hydrophobic core in the fibril [66]. Amino acid mutations therefore do not necessarily alter the stability of PrP but might have some local effects on the protein interactions which are required for oligomerization into fibrillar species. The exposure of hydrophobic regions in intermediate states during protein folding could increase the tendency towards aggregation, and subsequently initiate – at a certain stage – the misfolding cascade, which ultimately leads to disease. Hydrophobic interactions play a crucial role in the formation of β-sheets, as they bring fragments of a polypeptide chain in close proximity to each other [67]. Additionally, Kutznetsov and Rackovsky [68] showed that disease-promoting mutations in the human PrP^C^ had a statistically significant tendency towards increasing local hydrophobicity with a possible change in interactions between PrP molecules and/or between PrP and hypothetical cofactors that might initiate subsequent fibril formation.

## STABILITY AND CONVERSION PROPENSITY OF THE CARBOXY-TERMINAL PRION PROTEIN 

In contrast to the flexible N-terminal part of PrP^C^, structural details of the C-terminal globular domain are described for many species [28, 69, 70]. The overall folding of this region of PrP^C^ is very similar in most species. The superposition of various three-dimensional PrP^C^ structures, based on polypeptide backbone atoms of mammalian PrP^C^s, reveals only minor differences between the tertiary structures and major similarities in the secondary structures. These secondary structure elements have been the focus of a large number of conversion assays and computer-based molecular dynamics simulations [71-74]. The influence of certain amino acid residues within the α-helices or β-strands, as well as the deletion of secondary structure elements have been found to inhibit the conversion reaction in some experiments [74, 75] or have had an effect on the cellular localization of PrP [74]. 

Helices II and III are anti-parallel orientated and connected by a short loop. Their structural stability is supported by a disulfide bridge, which is parallel to both sheet-forming β-strands. The superpositioning and comparison of several mammalian PrP^C^ structures reveal that these α-helices and the β-sheet form the rigid core of the globular assembly. In contrast, helix I shows a more mobile positioning due to the long loop connections to corresponding secondary structure elements. The amino acid sequence composition of helix I is exceptional as it is the most hydrophilic α-helix of all known protein structures [76]. This possibly indicates a specific function in protein-protein interactions for this helix. In line with this assumption, recently published data show that helix I promotes aggregation of PrP but is not converted into β-strands [77].

A computational comparative analysis of PrP^C^ and Doppel, which is a structurally similar protein and which gene is located in the close vicinity of the prion gene, focussed on so-called chameleon sequences in both proteins [68]. Chameleon sequences are polypeptide fragments that can, experimentally, adopt both α-helical and β-strand conformations depending on the environmental conditions [78]. Other than PrP^C^, Doppel, which is not able to undergo misfolding, contains much shorter chameleon fragments. Interestingly, the most conserved part of PrP^C^ in all species contains an unusually long chameleon fragment, located in an unusually flexible sequence context between amino acid residues 114 and 125 [68]. Additionally, this 12-mer is a highly conformational variable polypeptide compared to other sequences of the same length. According to protein databank analysis, the mature PrP^C^ (without N- and C-terminal signal peptides) shows the highest conformational variability among all sequences that contain chameleon segments of 10-14 residues. The authors also found that the amino acids seen in the fragment between PrP residues 114-125 are involved in the formation of intermolecular complexes and possess high binding potential in other proteins. The results of this study concerning the highly conserved part of PrP, which contains the chameleon sequence, are in line with the results of Nguyen [79] and Zhang [80]. The researchers showed that peptides of this part of the PrP can adopt both α-helical or β-strand conformation. Interestingly, the peptide spanning aa 106-126 has shown to be neurotoxic [81]. 

Another exciting outcome of the computational examination by Kuznetsov and Rackovsky was that only helix I lacks a chameleon sequence in contrast to helix II and III of PrP^C^, which contain a chameleon hexamer (helix II) or a pentamer (helix III). The authors further show that helix I has a low β-strand propensity, especially when compared to helix II, which has a significant high propensity to β-strand conformation. Since helix I is not essential for prion infectivity [82], and it retains its α-helical conformation under a wide range of denaturing conditions [76, 83], it can be concluded that helix I does not unfold until the late states of structural transition, which occur in other parts of the PrP under the influence of global conformational rearrangements [76]. Due to the high conformational flexibility seen between residues 114-125 and with regards to the high β-strand propensity of helix II, it can be assumed that only moderate changes in the environmental conditions or interactions can induce misfolding of PrP and subsequent fibril formation.

The crystal structure of human PrP^C^ is the only example of a homodimeric PrP-structure [84]. In the homodimer, both α-helices I are in close anti-parallel orientation, allowing side chain contacts between the monomers. Other contacts in this homodimer are observed for the C-terminal parts of helices II, which form a new, short, anti-parallel β-sheet. This allows the reorientation of helices III for the dimer formation. The newly formed β-sheet is a possible initiation of α-β transition for the oligomerization of PrP [85].

A comparison of monomeric PrP structures with the prion-like protein Doppel [86] reveals a major deviation for the last two turns of helix II, where Doppel shows a strong kink. Consequently, the C-terminal two turns are positioned closer to the adjacent helix III. This could allow a more compact intramolecular interaction in Doppel, and it could explain why amyloidic misfolding of this protein is unknown.

Among various species of laboratory animals, the rabbit is a rare example of a TSE- resistant subject. Recently, the three-dimensional structure of rabbit PrP^C^ was determined by NMR spectroscopy (pdb entry code 2fj3). The aforementioned structural assumption – regarding helix II in Doppel – could be an explanation for the stability of rabbit PrP^C^, which also shows a C-terminal distortion of helix II (Fig. (**3**)).

The NMR structure of elk (*Cervus elaphus nelsoni*) PrP displays a species-specific characteristic in the region of aa 166-175. This loop links the second β-strand with the second α-helix. In contrast to other mammalian species, this loop is exceptionally well-defined in elk [87]. The homologous region is flexibly disordered in other mammalian PrP^C^ species such as mice, bovines and humans. By substituting certain amino acids in the corresponding region of the mouse PrP^C^, it has been shown that the rigidity of this loop results from the presence of asparagine at residue 170 in combination with threonine at residue 174. Although both amino acid side chains apparently do not interact with PrP^C^, they have an additional long range effect on α-helix III, which is better defined in the presence of asparagine and threonin than in wild-type mouse PrP^C^. Whether the rigid loop confers TSE susceptibility or pathological consequences remains to be examined [88].

## THE ROLE OF THE AMINO-TERMINAL REGION DURING PRION PROTEIN MISFOLDING

The amino terminal region of PrP^C^ residues 23-120 (which make up nearly the complete first half of the amino sequence of full-length matured PrP^C^) is unstructured [89] due to a high degree of main chain flexibility. Enzymatic degradation studies and transgenic mouse studies showed that the amino acids stretching from 23-89 are disposable in terms of generating infectious prions. Transgenic mice that express a truncated version of PrP^C^ that lacks the octarepeat region remain susceptible to prion infection [90], although disease progression is slowed down. The alteration of metal ion binding has been observed in human prion diseases [91]. This stretch harbours a region of octarepeat sequences with the ability to bind Cu^2+^ ions cooperatively [92], and it has been reported that incubation with copper ions at concentrations as low as 50 µM renders full length PrP^C^ PK resistant [93]. This is in line with the observation that preferential Cu^2+^ coordination by His96 and His111 induces beta-sheet formation in the unstructured amyloidogenic region of the prion protein [94].

This ability of PrP^C^ to bind metal ions is also seen in a non-vertebrate PrP-like molecule, termed "similar to prion protein" or StPrP [95]. Even though the metal ion binding site of StPrP consists of less amino acid repeats than the one seen in mammalian PrP^C^, it is able to bind Cu^2+^ as effectively as human PrP does. Using various PrP fragments and spectroscopic techniques, it has been shown that two Cu^2+^ ions bind to two binding sites centred at His111 and His96.

Transgenic mice expressing a prion protein with up to eight extra octapeptide repeat insertions suffered from a spontaneous non-infectious accumulation of PK resistant PrP in the brain [96], while transgenic mice expressing a bovine PrP with five octapeptide repeats displayed a reduced susceptibility to BSE infection [97]. The role of metal ion binding in these pathologies is still not fully determined. Due to the redox-properties of Cu^2+^-ions, oxidative stress is thought to induce prion misfolding. However, this assumption is still lacking solid experimental data. 

Cell-free conversion of a N-terminally truncated, ovine-mouse chimeric, bacterially expressed PrP by mouse-passaged BSE resulted in two PK resistant PrP^res ^fragments with a difference of about 1 kDa in their molecular mass [98]. In contrast to mouse- passaged BSE, mouse scrapie Me7, 22A or 87V induced a conversion into only one detectable PK resistant PrP^res^ fragment. These results show that the flexible N-terminal region might support a specific docking of PrP^C^ to PrP^Sc^. Depending on the structure of the PrP^Sc^ seed, the truncation of amino terminal parts of PrP^C^ can hinder either the binding of PrP^Sc^ in general – which leads to a decrease of conversion efficiency – or it inhibits a specific binding of PrP^Sc^, which results in the conversion of PrP^C^ into differently shaped and sized PK resistant PrP^res^ fragments. How Cu^2+^ and other metal ions influence the flexibility of the N-terminal part and subsequently the interaction between PrP^Sc^ and PrP^C^ *in vivo* and *in vitro* in a strain-specific fashion remains an interesting topic that has not yet been determined.

Many studies focus on the amino terminal part in order to define its relevance for the physiological function of PrP and its conversion into PrP^Sc^. Various aspects of transgenic mice with diverse deletions within the N-terminus, as well as truncated forms of recombinant PrP^C^, have been researched.

Even though PrP-knockout (PrP^0/0^) mice do not show remarkable deficits due to the lack of PrP^C^ expression, the deletion of residues 105-125 of PrP^C^ leads to neonatal lethality in transgenic mice [81]. The authors assume a neuroprotective function of PrP^C^, especially for the region between residue 105 and 125, which may be associated with the signal transduction in order to avoid cellular death; it may be converted to a neurotoxic signal by truncation of certain parts of PrP^C^. Neurotoxic effects, along with neurodegenerative disease, were also observed in transgenic mice expressing other N-terminally truncated forms of PrP^C^ [99]. 

Doppel, the downstream prion-like protein that shows similarities to the C-terminal domain of PrP^C^ but lacks the N-terminal part [100], also causes neurodegenerative dysfunction with massive Purkinje cell loss if overexpressed in transgenic PrP^0/0^ mice [100]. Since the reintroduction of a *prnp *transgene abrogates the disease [101], it is likely that PrP^C^ functions as neuroprotective molecule. It would be interesting to know if similar effects are provided by the reintroduction of *prnp* transgenes in those mice which encode different N-terminally truncated forms of PrP^C^. Distinct sequence motifs of the amino terminal part could also be examined for their possible neuro-protective function.

## THE NEUROTOXIC PRION PROTEIN FRAGMENT 106-126

Analysing different sections of the full length PrP, Forloni and co-workers [102] identified a peptide spanning aa residues 106-126 which displayed a neurotoxic effect on rat hippocampal neurons *in vitro.* Neurotoxicity could also be demonstrated *in vivo* [103]. This part of the prion protein is therefore commonly referred to as neurotoxic peptide. These toxic characteristic are restricted exclusively to cells which express PrP^C^ [104]. Because the prion fragment 106-126 shares many properties of PrP^Sc^, e.g. the ability to form β-structures and partially protease-resistant fibrils, it is seen as a model for molecular mechanisms in neurodegeneration caused by prion protein misfolding.

In the structural context, region 106-126 is in very close proximity to the first short β-strand. It contains the sequence 114-125, which – as mentioned before – has shown to be a chameleon motiv. Depending on the conditions, PrP106-126 can either adapt a metastable α-helical structure in organic solvents [105], or it self-assembles into amyloid-like fibrils in water [79]. The highly amyloigenic and hydrophobic palindrome AGAAAAGA is located between aa residue 113 and 120 of PrP 106-126. It is described as putative aggregation site [106], although this sequence requires its flanking parts to form fibrillar aggregates [107]. Within the amyloidogenic sequence, Jobling [108] replaced the hydrophobic amino acids with hydrophilic serines. These mutations decreased the β-sheet structure and, in correlation with this, abolished neurotoxicity. Due to the alterations in the secondary structure, the aggregation and fibrillogenic properties of PrP 106-126 also changed. In contrast to the correlation of amyloidogenicity and neurotoxicity, Bergström [109] showed that the oxidation of human PrP 106-126 methionines reduced the propensity to form amyloid firbrils but at the same time increased cytotoxicity. A similar result was shown in another experiment with a mutant form of PrP 106-126. The replacement of glycine at residues 114 and 119 by alanine led to the inability of the peptide to build fibrils but it nevertheless increased cytotoxicity [110]. The authors assume that by diminishing fibril formation, the peptides could be available as soluble oligomers, which are more toxic than larger amyloid fibrils [109]. 

## CONCLUDING REMARKS

Protein misfolding remains a conundrum, although there are plenty of research methods that aim to resolve this question. Structural aspects of the prion protein, including the amino acid sequence, secondary structural elements and post translational modifications have been taken into consideration with regard to the conformational transition of the protein. The ability of prions and other misfolding proteins to adapt more than one stable conformation has brought up numerous questions concerning the mechanisms of protein folding, unfolding and misfolding. There is no doubt that not only the intrinsic properties of a polypeptide chain determine its three dimensional structure, but also the multiple environmental influences such as the cellular milieu which contribute to the conformation of a protein.

The development and establishment of new research methods and tools over the last decades have opened up possibilities to investigate and understand the nature of protein folding and misfolding. Discovering the mechanisms behind this complex process of conformational transition will establish new prospects for combating the increasingly common and most devastating protein misfolding diseases.

## Figures and Tables

**Fig. (1):**
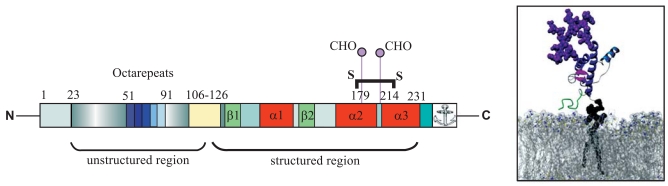
Schema of the cellular mouse prion protein (left): the N-terminal signal sequence (aa 1-22) is removed during posttranslational processing. The unstructured region (aa 23-121) harbors five octarepeats, which function as binding site for bivalent ions, such as Cu^2+^. The neurotoxic peptide (aa 106-126) is show in yellow. The globular domain consists of two very short β-strands (aa 128 to 130 and aa 160 to 162; light green) and three α-helices (aa 143 to 153, aa 171 to 192, aa 199 to 226; red). The disulfide bond between aa 179 and aa 214 stabilizes the three dimensional structure of the protein. Two glycosylation sites are located at aa 180 and 196, where oligosaccharide chains are linked to the polypeptide chain. A GPI anchor (aa 231) attaches the protein to the cell membrane, as shown in the right Picture. DeMarco, M.L., and Daggett, V. (2005). *Comptes Rendus Biol*., **328**, 847-862.

**Fig. (2):**
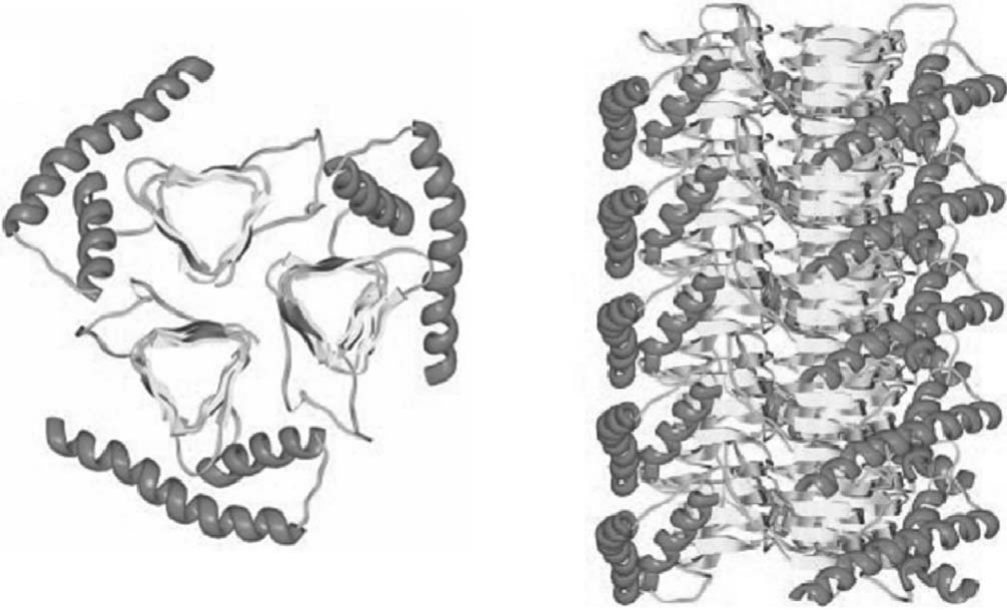
Model of a three dimensional structure for PrP^Sc^: the β-sheets fold into β-helices. Three of these β-helix molecules form the basic unit for a PrP^Sc^-fibril (right). Govaerts, C., Wille, H., Prusiner, S.B., and Cohen, F.E. (2004). *Proc. Natl. Acad. Sci. USA*, **101**, 8342-8347.

**Fig. (3):**
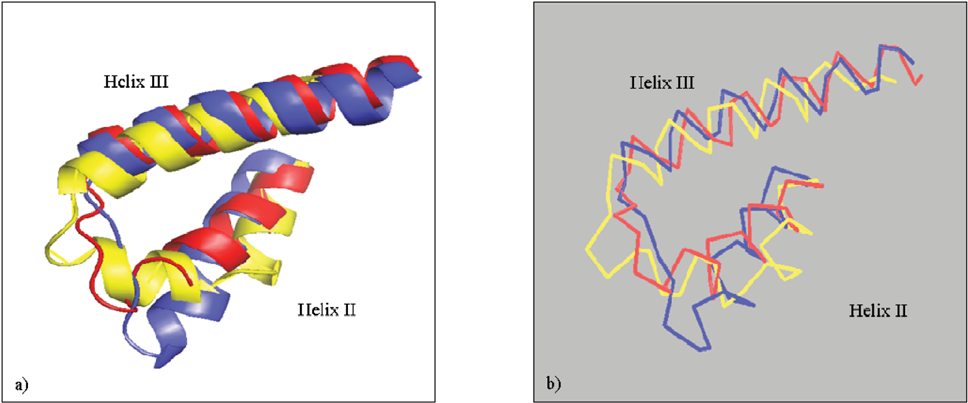
**a**) Cartoon plots of helices II and III of superimposed structures of rabbit-PrP (red), mouse-PrP (blue) and mouse-Doppel (yellow). The C-terminal turn of helix-II and the adjacent loop connection of rabbit-PrP deviate significantly to mouse-PrP and follow more the conformation of the kinked helix II of mouse-Doppel. **b**) Cα-tracing of helices II and III of PrP from rabbit-PrP (red), mouse-PrP (blue) and mouse-Doppel (yellow). Superposition based on Cα-positions with PrP-mouse as a target.
